# Potential Impact of Rapid Multiplex PCR on Antimicrobial Therapy Guidance for Ventilated Hospital-Acquired Pneumonia in Critically Ill Patients, A Prospective Observational Clinical and Economic Study

**DOI:** 10.3389/fcimb.2022.804611

**Published:** 2022-04-13

**Authors:** Florian Guillotin, Cécile Poulain, Benjamin Gaborit, Marwan Bouras, Raphaël Cinotti, Karim Lakhal, Mickael Vourc’h, Bertrand Rozec, Karim Asehnoune, Marie-Anne Vibet, Valéry-Pierre Riche, Sophie-Anne Gibaud, Lise Crémet, Antoine Roquilly

**Affiliations:** ^1^Nantes Université, CHU Nantes, Anesthesie Réanimation, INSERM, Center for Research in Transplantation and Translational Immunology, Nantes, France; ^2^Service de Maladies Infectieuses et Tropicales et CIC 1413, CHU Nantes, Nantes, France; ^3^Service d’ Anesthésie-Réanimation, Hôpital Nord Laënnec, CHU Nantes, Nantes, France; ^4^Plateforme de Méthodologie et Biostatistique, CHU de Nantes, Nantes, France; ^5^Cellule Innovation - Département Partenariat et Innovation - Direction de la Recherche, CHU de Nantes, Nantes, France; ^6^Service de Bactériologie-Hygiène, Pôle de Biologie, CHU de Nantes, Nantes, France

**Keywords:** pneumonia, biomolecular diagnosis, empiric treatment, intensive care unit, hospital-acquired pneumonia

## Abstract

**Objectives:**

To investigate the potential impact of the syndromic multiplex FilmArray^®^ Pneumonia *plus* Panel (FAPP) on the antimicrobial treatment guidance of patients with ventilated hospital-acquired pneumonia (VHAP).

**Methods:**

Respiratory fluids from 100 adult patients with VHAP, receiving invasive mechanical ventilation in three intensive care units from one French university hospital, were tested prospectively using FAPP. Conventional cultures were performed in parallel as routine practice. Clinicians were left blinded to the FAPP results. Antimicrobial therapies based on FAPP results were simulated by independent blinded experts according to a predefined algorithm and compared to 1) those prescribed in practice according to local guidelines (real-life), and 2) those that complied with the international ERS/ESICM/ESCMID/ALAT recommendations. The primary endpoint was the number of days of broad-spectrum antimicrobial therapy. Secondary endpoints were the rates of microbiological treatment failure and cost-effectiveness ratio.

**Results:**

The predicted median duration of broad-spectrum antibiotics was 0 [0-1.25] day in the FAPP-based simulation, versus 2 [0-6] days in real-life (p<0.0001) and 2 [2-3.25] days in the recommendations-based simulation (p<0.0001). Treatment failure was predicted in 3% of cases with FAPP results versus observed in 11% in real-life (p=0.08) and 6% with recommendations-based simulation (p=0.37). The incremental cost-effectiveness ratio was 1 121 € [-7021; 6794] to avoid one day of non-optimized antimicrobial therapy.

**Conclusions:**

Our results suggest that using FAPP in patients with VHAP has the potential to reduce the use of broad-spectrum antimicrobial therapy without increasing the risk of microbial treatment failure.

## Introduction

Ventilated hospital-acquired pneumonia (VHAP) is a frequent complication and one of the leading causes of antibiotic use in intensive care units (ICU) (de Miguel-Díez et al., 2017). VHAP is associated with prolonged duration of mechanical ventilation and hospital stay and generates elevated healthcare costs (Safdar et al., 2005; Melsen et al., 2013; Ohannessian et al., 2018). Moreover, VHAP caused by difficult-to-treat multidrug-resistant (MDR) bacteria are increasingly prevalent (EU-VAP Study Investigators et al., 2013) and result in significant morbidity and mortality (Bassetti et al., 2017).

The identification of pathogens in respiratory fluids requires two to three days, and it is recommended to initiate an empirical antimicrobial treatment immediately after the collection of respiratory fluids. A broad-spectrum empirical antimicrobial therapy is recommended for VHAP considered at risk of MDR bacteria to decrease the risk of empiric treatment failure. Validated risk factors for MDR bacteria are a prior infection/colonization with MDR bacteria, any antimicrobial therapy in the preceding 90 days, and a late-onset VHAP, i.e., within or after the fifth day of hospitalization (Braykov et al., 2014; Kalil et al., 2016). However, using these criteria leads to unnecessary broad-spectrum antimicrobial therapy in up to 60% of patients with VHAP (Monard et al., 2020), which substantially increases the risk of selecting resistant Gram-negative bacilli (Armand-Lefèvre et al., 2013) without providing any individual benefit. While the delay to optimal empirical antimicrobial treatment is associated with mortality in septic patients, it is challenging to balance the benefit/risk ratio between treatment failure and unnecessary broad-spectrum treatment (Torres et al., 2017; Leone et al., 2018; Roquilly et al., 2020).

Rapid multiplex molecular tests, which can reduce the turnaround time for bacterial identification and antimicrobial susceptibility testing, are now available at the bedside (Huang et al., 2013; Cambau et al., 2017). However, it remains unclear if rapid PCR tests should be used to decrease the risk of treatment failure or prevent unnecessary broad-spectrum antimicrobial therapy. In a prospective cohort of 100 patients with ventilated hospital-acquired pneumonia (VHAP), we found that the FilmArray^®^ Pneumonia *plus* Panel (FAPP) enhanced the positivity rate of conventional diagnostic testing, with increased recognition of coinfections and reduced time-to-results by more than 48 hours (Buchan et al., 2020; Crémet et al., 2020). In this study, using the clinical data of the same cohort, our first objective was to determine the theoretical impact of FAPP results on antimicrobial therapy for VHAP in patients receiving invasive mechanical ventilation. Our second objective was to assess the cost-effectiveness of this new molecular test.

## Methods

### Ethics

A local ethic committee approved this study (GNEDS, Nantes, France). FAPP occurred using a research without prior consent model, and patients or next-of-kin were informed retrospectively of their participation in this study and of the possibility of being opposed to the use of data.

### Study Design

This prospective observational study was conducted in 3 ICUs of a tertiary hospital in France between October 2018 and January 2020. One bacteriological laboratory performed centralized analysis for the three ICUs.

### Bacteriological Analysis

Each respiratory specimen was analyzed by standard culture and using the FAPP panel as soon as possible, and time-to-results were prospectively collected. As previously described, the bacterial culture was performed and interpreted independently of the multiplex PCR (Crémet et al., 2020). Physicians in charge of the patients were kept blinded to the FAPP results. The resistance genes and all bacteria detected with FAPP, independently from their bin level (DNA copies/mL), and the gram stain examination, were considered for the FAPP-based antibiotic simulation.

### Study Population

Adult mechanically-ventilated patients were included when collecting a respiratory fluid sample for the first VHAP diagnosis. Patients were not included in case of pregnancy, legal protection, predicted survival inferior to 48 hours.

### Definitions

VHAP was defined on European guidelines as a new or persistent radiological pulmonary infiltrates without another apparent cause combined with two clinical signs among fever, purulent endotracheal secretions, hyperleukocytosis, or leukopenia, and increasing oxygen requirements (Torres et al., 2017). Respiratory specimens were obtained before modification or initiation of new antimicrobial therapy by bronchoalveolar lavage or endotracheal aspiration.

Broad-spectrum antimicrobial therapy was defined as any molecules with activity against *P. aeruginosa*, including piperacillin-tazobactam, cefepime, ceftazidime, ciprofloxacin, and carbapenems with anti-pseudomonas activity (Roquilly et al., 2016; Torres et al., 2017). Narrow-spectrum antimicrobial therapy was considered if antimicrobial therapy had no activity against *P. aeruginosa* such as cefotaxime, ceftriaxone, amoxicillin-clavulanate, and ofloxacin.

Empiric antimicrobial therapy was considered optimal when all pathogens found in culture were susceptible to treatment, and no alternative with a narrower spectrum was available. Un-necessary broad-spectrum treatment was considered if all pathogens were susceptible to narrow-spectrum antimicrobial agents. Microbial treatment failure was defined as a positive culture with one or more bacterial pathogen resistant to the empiric antimicrobial therapy.

### Study Endpoints

The primary endpoint was the number of days of broad-spectrum antimicrobial therapy to treat the first pneumonia episode.

Secondary endpoints were 1) the rate of microbiological treatment failure, 2) the total number of days of narrow-spectrum antimicrobial therapy and carbapenems with anti-pseudomonas aeruginosa activity, 3) the difference of rate of non-optimal empiric antimicrobial therapy, and 4) the cost-effectiveness ratio.

### Antimicrobial Therapy Groups

Three empiric antimicrobial therapies were compared: one was the real-life treatment, i.e., the treatment that was administered, and two were simulated by two groups of two medical experts in critical care or infectious diseases after careful reviewing of the medical chart: one was proposed based on international ERS/ESICM/ESCMID/ALAT recommendations (Torres et al., 2017), and the other based on FAPP results. In case of disagreement between both experts, a college of six physicians blindly reviewed the case in order to determine the simulated treatment. In order to limit the risk of bias, the experts were kept blinded by the real-life treatment.

Real-life treatment. According to our local standard of care, amoxicillin-clavulanate was recommended up to the 10th day of hospitalization if the patient was admitted for trauma or brain injury and had no prior antimicrobial treatment of more than 48h (Crémet et al., 2020). Otherwise, a narrow-spectrum antimicrobial treatment was recommended up to 5 days of hospitalization. De-escalation was recommended when possible after bacterial identification. A total of 7 days of antimicrobial therapy was recommended, without biomarker-guided adaptation of the therapy.

Recommendations-based treatment. Broad-spectrum empiric antimicrobial therapy was considered in the case of MDR bacteria risk factors (late-onset VHAP, recent history of antimicrobial therapy, or prior infection/colonization with MDR bacteria). De-escalation or optimization was simulated immediately after the bacterial identification by standard culture. The time of adaptation was considered as the time of biological validation.

FAPP-based treatment. Antimicrobial stewardship based on the pathogens and resistance genes identified with the multiplex panel was *a priori* developed by intensivists, infectious disease specialists, and microbiologists (**Supplementary Table S1**). De-escalation or optimization was simulated immediately after the bacterial identification by standard culture. The time of adaptation was considered as the time of biological validation. We have chosen not to consider the results of FAPP for de-escalation.

### Data Collection

Clinical data were prospectively collected and anonymized: age, length of stay (LOS), cause of admission, IGSII, vasopressors therapy, PaO2/FiO2 ratio, risk factors for MDR gram-negative bacilli and Methicillin-Resistant *Staphylococcus aureus*, in-ICU and in-hospital mortality, antimicrobial therapy, second episode of VHAP.

### Medico-Economic Evaluation

We conducted a cost-effectiveness analysis (CEA) comparing the real-life antimicrobial therapy to the simulated antimicrobial therapy using the FAPP results to evaluate the efficiency of FAPP. CEA was carried out during a time horizon that includes the resolution of the infectious episode and from the hospital perspective. Indeed, healthcare resources consumption related to an infection and its management are included in the diagnostic related group (DRG). Effectiveness was evaluated on the optimization of antibiotic prescription: number of days of ineffective empirical antibiotic or with unnecessary broad-spectrum. Considered costs are those related to antimicrobial therapy and FAPP use. We did not consider costs related to culture tests because they are made in all cases. Costs were expressed in euros. Data were obtained using the patient’s medical chart.

### Statistical Analysis

No information was available to estimate the reduction of broad-spectrum antimicrobial therapy with FAPP. We decided to include 100 patients for feasibility. We performed a statistical analysis following a hierarchical and sequential strategy to test the primary and secondary hypotheses. The hypotheses involved two hypotheses ranked according to their clinical relevance: first, the comparison between the recommendations-based simulation and FAPP-based treatment. Then, the comparison between the real-life and FAPP-based treatments. The effect of such a procedure is that no confirmatory claim can be based on variables that rank lower than or equal to that variable whose null hypothesis was the first that could not be rejected (Watanabe and Takahashi, 2006; Huque and Alosh, 2008). The type I error rate remained controlled at 5% for each test. The secondary endpoints were tested using the same procedure. When the first hypothesis was not significant, results of the second hypothesis were presented using Bonferroni’s correction for adjusting p-values.

Continuous variables were presented as medians and quartiles (means and standard deviations were used when medians and quartiles were equal to 0), and categorical data were presented as exact numbers and percentages. The statistical test was a paired Student test or a Wilcoxon paired test for continuous variables if the normality conditions were not satisfied. For binary variables, the McNemar test was used for paired comparisons. The exact McNemar test with difference in proportions will give an exact test of paired binary responses, with compatible confidence intervals on the difference in proportions.

## Result

### Characteristics of the Population

One hundred patients were included. Demographic characteristics are presented in **Table 1**. The most frequent causes of admission were trauma (42%) and medical conditions (32%). For 96 patients, pneumonia occurred under invasive mechanical ventilation initiated for another reason, and 4 patients were intubated for a HAP. The median VHAP onset was 5 [3-9] days, and the median PaO2/FiO2 ratio at the time of HAP was 135 [102-195]. At the time of HAP diagnosis, FAPP yielded positive results with significant levels (i.e., ≥ 10^4^ bin in BAL and ≥ 10^5^ bin in ETA for semi-quantified bacteria) in 82/100 patients (Crémet et al., 2020). Culture identified one or more bacteria in 73/100 patients. Half of the specimens were concordant for the bacterial identification (43/76 (56.6%) BAL and 46/82 (56.1%) ETA). In most of the discordant specimens (23/33 (69.7%) BAL and 21/36 (58.3%) ETA), FAPP identified one supplemental bacterial pathogen. Bacteria detected by FAPP and standard cultures are described in the **Supplementary Figure S1**.

**Table 1:** Population Characteristics.

Characteristics	Study population (n=100)
Age, years, *median (25-75 percentile)*	57 [38.25-64.25]
Female, *N (%)*	19 (19)
Reason of admission in ICU, *N (%)*	
Trauma	42 (42)
Burn	7 (7)
Emergency surgery	5 (5)
Scheduled surgery	13 (13)
Medical	32 (32)
Other	1 (1)
Mechanically ventilated before pneumonia onset	96 (96)
VHAP onset, hospital days, *median (25-75 percentile)*	6 [3.75-10]
VHAP onset, ICU days, *median (25-75 percentile)*	5 [3-9]
Medical history of ESBL colonization or infection, yes	2 (2)
Risk factors for MRSA colonization, yes	6 (6)
Antibiotics exposure in the 90 days before inclusion, yes	40 (40)
VHAP severity	
SAPS II	43.64 [32-54.25]
PaO2/FiO2	135 [102-195]
Septic shock	21(21%)
In-ICU mortality, *N (%)*	21 (21)
28-days mortality, *N (%)*	17 (17)

ESBL, Expended spectrum beta-lactamases; MRSA, Methicillin-resistant Staphylococcus Aureus; HAP, hospital-acquired pneumonia; ICU, intensive care unit.

### Primary Outcome

**Figure 1:**
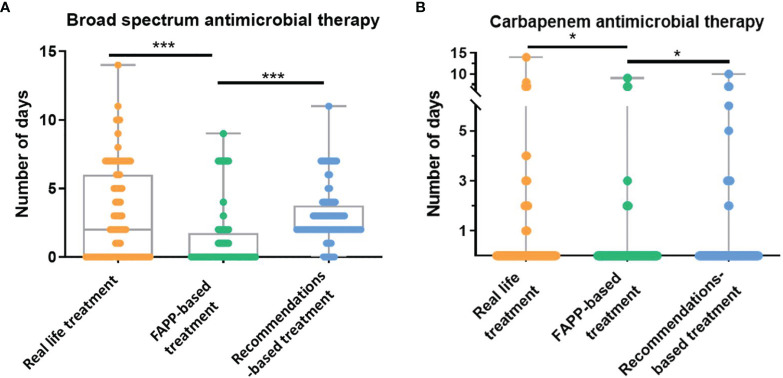
Duration of broad-spectrum antimicrobial therapy. **(A, B)**. Numbers of days of **(A)** broad-spectrum antimicrobial therapy (activity against P. aeruginosa), and **(B)** carbapenems with anti-pseudomonas aeruginosa activity in real-life, in the FAPP-based simulated treatment, and the recommendations-based simulated treatment. FAPP, FilmArray^®^ Pneumonia plus Panel. *p < 0.01, ***p < 0.001.

The median duration of broad-spectrum antimicrobial therapy was 0 [0-1.2] day in the FAPP-based simulation as compared to 2 [0-6] days in real-life and 2 [2-3.2] days in the recommendations-based simulation (p<0.0001 *vs.* two others) (**Figure 1**). The reduction in the duration of broad-spectrum antimicrobial therapy with FAPP remained significant in the trauma, medical and surgical subgroups (see **Supplementary Table S2**).

### Secondary Outcomes

**Figure 2:**
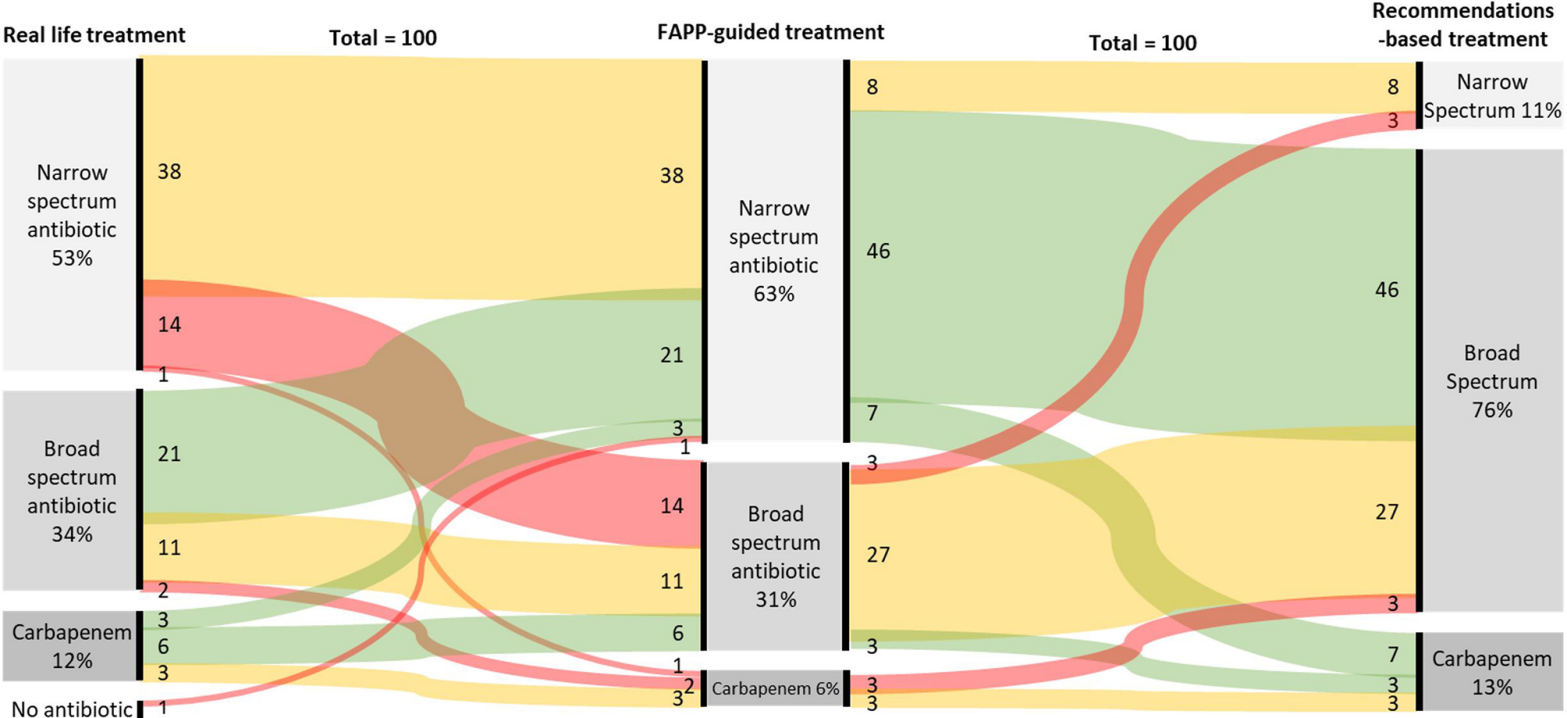
Sankey chart of real-life empirical treatments implemented without FAPP (left column), antimicrobial therapies simulated with the results of FAPP (Middle column), and antimicrobial therapies simulated by following the actual recommendations (right column). Red lines stand for antibiotic escalation, green for de-escalation, and yellow for no changes.

A broad-spectrum empirical therapy would have been administrated in 37% of patients based on the FAPP-guided simulation *vs.* 47% in real-life and 88% if international recommendations had been followed (p<0.01 *vs.* real-life and FAPP, **Table 2**). The antimicrobial therapies are described in **Figure 2** and **Supplementary Figure S2**. In the FAPP-based strategy, carbapenems with anti-pseudomonas activity were administrated for 0.3±1.4 days *vs* 0.5±1.7 days in the recommendations-based simulation (p=0.03) and 0.7±2.1 days for the real-life treatment (p=0.01). The FAPP-based simulated antimicrobial therapy was different from real-life treatment in 48% of the patients (**Figure 2**). The total number of days of narrow-spectrum therapy was 6 [1-7] days in the FAPP-based simulation *vs.* 4 [0-5] days in the recommendations-based simulation and 4 [0-7] days in real-life (p<0.01 *vs.* real-life) (**Table 2**).

**Table 2:** Primary and secondary outcomes.

	FAPP based treatment	Recommendation-based treatment			Real-life treatment		
	N=100 patients	N=100 patients	difference with FAPP (95% CI)	P Values ***	N=100 patients	difference with FAPP (95% CI)	P Values ***
							
Broad Spectrum treatment^#^
*Yes, (%)*	36 (36%)	92 (92%)	-56 [-66; -45]	<0.0001	58 (58%)	-22 [-26; -4]	<0.0001
*Duration, days* *	0 [0, 1.2]	2 [2, 3.2]	1.7 [1.40; 2]	<0.0001	2[0,6]	1.7 [1.2; 2.3]	
Carbapenems with anti-pseudomonas activity,							
*Yes, (%)*	7 (7%)	12 (12%)	-3 [-12; 2]	0.18	18 (18%)	-11 [-20; -3]	0.004
*Duration, days* **	0.3 ± 1.4	0.5 ± 1.7	0.2[0; 0.4]	0.03	0.7 +/- 2.1	0.4 [0.1; 0.7]	0.01
Narrow spectrum treatment^##^							
*Yes, (%)*	81 (81%)	67 (67%)	14 [5; 22]	<0.001	70 (70%)	11 [0; 22]	0.05
*Duration, days* *	6 [1, 7]	4 [0, 5]	-1.5 [-1.8;-1.2]	<0.0001	4[0, 7]	-0.9 [-1.5;-0.3]	0.002

Comparison of the duration of narrow-spectrum, broad-spectrum and carbapenem antibiotic therapy during the entire course of pneumonia treatment, including empirical and adapted antimicrobial treatment.

^#^Broad-spectrum antimicrobial therapy was defined as any molecules with activity against P. aeruginosa.

^##^Narrow-spectrum antimicrobial therapy was considered if antimicrobial therapy had no activity against P. aeruginosa.

FAPP, FilmArray^®^ Pneumonia plus Panel. *Median [IQR], **For carbapenems, medians [IQR] were 0 [0-0]. Hence means and standard deviations are presented.***P Values for comparison with FAPP-based treatment.

Out of the 100 included patients, 67% had an optimal empiric antimicrobial therapy in the FAPP-based simulation, 19% in the recommendations-based simulation (p<0.0001), and 61% in real-life (p=0.38) (**Table 3**). Microbiological failure was observed in 3% of the FAPP-based simulation of treatment vs. 6% of the recommendations-based simulations (p=0.37) and 11% of the in-real-life treatment (p=0.08).

**Table 3:** Adequation of the spectrum of empirical antimicrobial therapy with pathogens found in culture.

	FAPP-based treatment	Recommendations-based			Real-life treatment		
	Percentage of patients n=100	Percentage of patients n=100	OR [95%CI]	P values *	Percentage of patients n=100	OR [95%CI]	P values *
Optimal treatment	67%	19%	0.25 [0.1; 0.4]	<0.0001	62%	1.5 [0.9; 3.2]	0.38
Treatment Failure	3%	6%	0.25 [0.005; 2.5]	0.37	11%	0.2 [0.01; 1.1]	0.08*
Un-necessary broad-spectrum	30%	76%	0.09 [0.03;0.24]	<0.0001	27%	1.3 [0.5; 3.3]	0.68

Optimal treatment was considered when all pathogens found in culture were susceptible to treatment, and no alternative with a narrower spectrum was available. Un-necessary broad-spectrum treatment was considered if all pathogens were susceptible to narrow-spectrum antimicrobial agents. Microbial treatment failure was defined as a positive culture with one or more bacterial pathogen resistant to the empiric antimicrobial therapy. Comparison of the efficiency of empiric antimicrobial therapies by a Mc-Nemar test. *p-values for comparison with FAPP-based treatment adjusted for multiple tests using Bonferroni’s method.

### Economic Analysis

As shown in **Table 4**, the cost to avoid one day of non-optimized antibiotics was 1 121 € [-7021; 6794], indicating that FAPP utilization is more expensive but more effective than the guidance of empirical antimicrobial therapy based on local protocol or international recommendations. The sensitivity analysis conducted on the FAPP price reveals that the strategy always generates an additional cost due to a higher mean cost of probabilistic antimicrobial therapy in the FAPP group (considering an extreme FAPP price of 0 € the cost to avoid one day of non-optimized antibiotics still remain positive: 6 € to avoid one day of non-optimized antimicrobial therapy).

**Table 4:** Medico-economic analyses.

	Standard bacterial culture alone	FAPP and bacterial culture
Total antibiotics cost per patients (a) (€)	3431,66	3617,36
Mean antibiotics cost per patients (€)	36,12	38,07
Min antibiotics cost (€)	3,30	5,4
Max antibiotics cost (€)	146,39	541,5
Standard derivation (€)	21,86	54,85
Total (FAPP) (b) (€)	0	32319
Mean (FAPP) per patient (€)	0	340,2*
Total (a+b) (€)	3431,66	35936,36
Effectiveness (day of non-optimized antimicrobial therapy)	112	83
Incremental Cost-Effectiveness Ratio (€ to avoid one day of non-optimized antimicrobial therapy)	$\frac{35936,36-3431,66}{83-112}=1121$	

*French tariff per test for conventional or multiplex quantitative real-time PCR for ≥ 10 primer pairs (DNA/RNA), from positif list for medical biology act (“Référentiel des actes innovants hors nomenclature” RIHN, Reference Document for Innovative Procedures).

^a^Total antibiotics cost per patients.

^b^Total (FAPP).

## Discussion

In this prospective observational study, we showed that as compared to guidance by optimized local protocol or by international recommendations, FAPP has the potential to reduce the number of days of broad-spectrum antimicrobial therapy without increasing the risk of treatment failure in critically ill patients with VHAP.

The use of FAPP in this study would have led to a reduction of consumption of broad-spectrum antibiotics and fewer days under carbapenems with anti-pseudomonas activity. Even if the study was underpowered to demonstrate a superiority of FAPP-based empiric antimicrobial therapy on the risk of treatment failure, our study suggested that the collective benefit of reducing unnecessary broad-spectrum treatment was not at the price of an increase of the individual. This strategy made it possible to achieve the challenge of limiting broad-spectrum antimicrobial therapies to the infections that require such a treatment.

In our study, only 67% of empiric antimicrobial therapies were considered optimal in the FAPP-guided simulation. This low percentage could be explained by the study protocol established to guide empiric antimicrobial therapy based on PCR results. We recommended the prescription of narrow-spectrum antibiotics in negative multiplex PCR and negative Gram stain examination. We made this choice because of the possibility of false-negative results with PCR, but this led to the prescription of antibiotics if the culture was finally negative. This choice can certainly be adapted to each situation; notably, no treatment could be proposed for PCR negative results and negative Gram stain examination in the absence of VHAP severity or when the VHAP diagnosis is uncertain.

We estimated that the implementation of FAPP would increase the medical cost by 1 121 € to avoid one day of non-optimized empiric antibiotics. This estimation was based on considering that all patients with VHAP would have FAPP. With the currently described approach, the additional cost associated with the use of FAPP represented 0.6% of the total costs of hospitalization estimated at €55 000 per patient in this population. It is thus theoretically possible to increase FAPP efficiency by limiting its indications to patients at risk of BMR bacteria. Moreover, we only considered the impact of FAPP on the cost of antimicrobial therapy. However, we identified MDR bacteria requiring patient isolation in 8 episodes of VHAP, out of which 7 had no history of resistance. Since we have already demonstrated that the time-to-result was reduced by two days compared to conventional cultures, FAPP may result in earlier isolation of patients with MDR bacteria, thus reducing the risk of colonization of other patients.

We found that the use of FAPP was still 6€ more expensive, even if we consider the use of the kit as free. This is because the price of antibiotics is not correlated to their spectrum. Thus the use of some narrow spectrum antibiotics is more expensive than the use of some broad spectrum antibiotics.

### Limits

There was no difference in unnecessary broad-spectrum antibiotics between our study’s FAPP-based and real-life treatments. This could be explained by the antimicrobial stewardship implemented in our center. As previously published (Roquilly et al., 2016), HAP and VAP are treated with amoxicillin-clavulanate within the first ten days of hospitalization in traumatic or brain-injured patients. This local standard of care was implemented to decrease the consumption of broad-spectrum antibiotics and could have minimized the drop in consumption of broad-spectrum antibiotics with FAPP.

The study was conducted before the covid-19 pandemic, and FAPP does not currently detect the SARS-cov2. The advantage of FAPP is its ability to diagnose better coinfections than standard cultures (Crémet et al., 2020), which could be helpful in covid-19 patients who frequently develop VAP in case of prolonged mechanical ventilation. A study has already investigated the efficacy of FAPP in 43 critically ill patients with covid-19, showing sensitivity, specificity, positive and negative predictive values of 95%, 99%, 82%, and 100%, respectively (Caméléna et al., 2021).

There were only eight patients with infections caused by MDR bacteria in this monocentric study. The reduction of broad-spectrum antimicrobial therapies by FAPP could be lower in ICU with a high rate of MDR bacteria.

Finally, the observational design of our study did not permit us to draw definitive conclusions since the application of FAPP-based treatment in clinical practice could be challenging if clinicians decide to follow international recommendations rather than FAPP-results.

In this prospective observational cohort, our results suggest that a rapid syndromic multiplex PCR to identify pathogens and resistance genes in lower respiratory tract specimens has the potential to reduce the use of unnecessary broad-spectrum antimicrobial therapy without increasing the risk of treatment failure. Confirming our results in further interventional studies will be needed before making any recommendations for clinical practice.

## Data Availability Statement

The datasets presented in this article are not readily available because Clinical data were prospectively collected and anonymized. Requests to access the datasets should be directed to florian.guillotin@chu-nantes.fr.

## Ethics Statement

The studies involving human participants were reviewed and approved by GNEDS, Nantes, France. Written informed consent for participation was not required for this study in accordance with the national legislation and the institutional requirements.

## Author Contributions

FG, CP, SG, LC and AR contributed to conception and design of the study. FG and CP organized the database. FG, CP, SG, LC, BR, KL, BG, MB contribute to inclusions in the study. M-AV and V-PR performed the statistical analysis. FG wrote the first draft of the manuscript. M-AV and V-PR wrote sections of the manuscript. All authors contributed to manuscript revision, read, and approved the submitted version.

## Funding

This study was funded by BioMerieux, who had no role in patient recruitment, analyses of the data, drafting, and decision to submit or revise the manuscript.

## Conflict of Interest

Author BG was employed by Service de Maladies Infectieuses et Tropicales et CIC 1413.

The remaining authors declare that the research was conducted in the absence of any commercial or financial relationships that could be construed as a potential conflict of interest.

## Publisher’s Note

All claims expressed in this article are solely those of the authors and do not necessarily represent those of their affiliated organizations, or those of the publisher, the editors and the reviewers. Any product that may be evaluated in this article, or claim that may be made by its manufacturer, is not guaranteed or endorsed by the publisher.
