# Supplementary material for: Potential Impact of Rapid Multiplex PCR on Antimicrobial Therapy Guidance for Ventilated Hospital-Acquired Pneumonia in Critically Ill Patients, A Prospective Observational Clinical and Economic Study
Source: Front Cell Infect Microbiol. 2022 Apr 13;12:804611. doi: 10.3389/fcimb.2022.804611 (PMC9043525; doi:10.3389/fcimb.2022.804611)
Supplement: Supplementary file 1 [file DataSheet_1.pdf]

Potential impact of Rapid Multiplex PCR on antimicrobial therapy guidance for ventilated hospital-acquired pneumonia in critically ill patients, a prospective observational clinical and economic study

Florian Guillotin, Cécile Poulain, Benjamin Gaborit, Marwan Bouras, Raphael Cinotti, Karim Lakhal, Mickael Vourc'h, Bertrand Rozec, Karim Asehnoune, Marie-Anne Vibet, Valéry-Pierre Riche, Sophie Gibaud, Lise Crémet, Antoine Roquilly

Supplementary Material

Figure S1. FAPP results compared to culture results for bacterial detection…………………….2

Figure S2: Sankey chart of real-life empirical treatments implemented without PCR (left column), antimicrobial therapies simulated with the results of PCR (Middle column), and antimicrobial therapies simulated by following the actuals international guidelines (right column)……………………………………………………………………………………………..3

Table S1: Antimicrobial stewardship developed to guide therapy on the basis of FAPP result…..5

Table S2: Subgroup analysis of the primary outcome……………………………………………6

Figure S1. FAPP results compared to culture results for bacterial detection

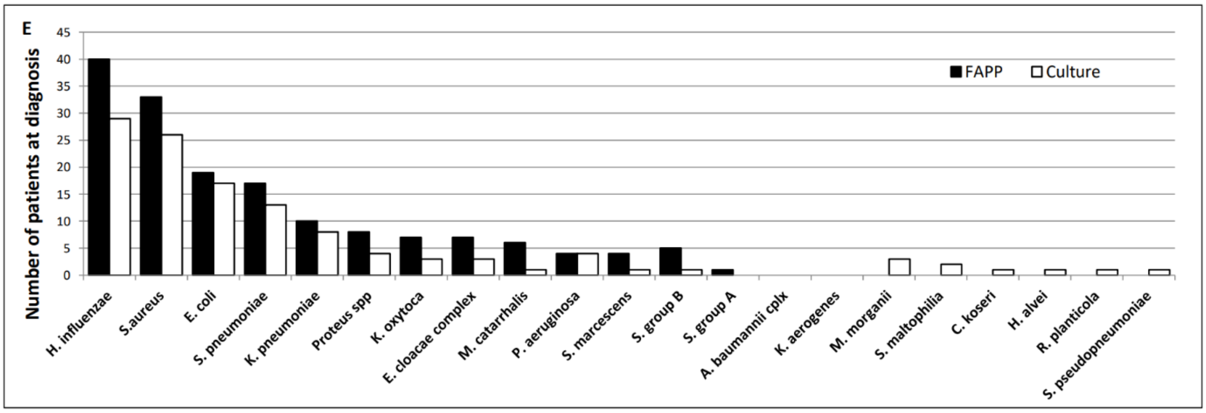

FAPP *FilmArray® Pneumonia plus Panel*

Figure S2: Sankey chart of real-life empirical treatments implemented without PCR (left column), antimicrobial therapies simulated with the results of PCR (Middle column), and antimicrobial therapies simulated by following the actuals international guidelines (right column).

The lines are colored in red for antibiotic escalation, green for de-escalation and yellow for no changes.

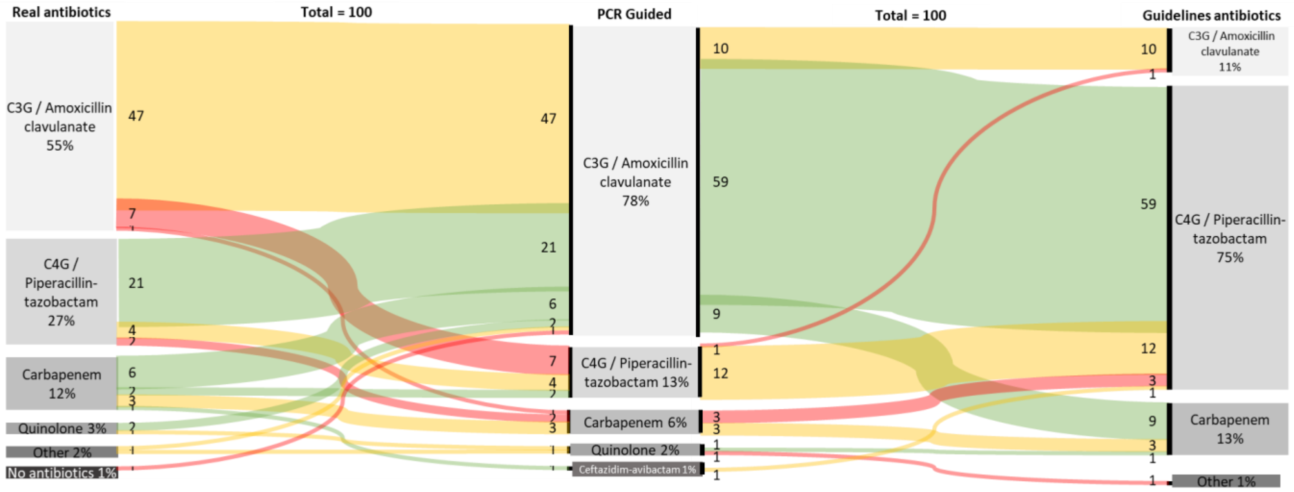

PCR guided: *FilmArray® Pneumonia plus Panel*-guided

Table S1: Antimicrobial stewardship developed to guide therapy on the basis of FAPP results.

Multiplex PCR results		Empiric antibiotherapy recommended	
Positive for Antimicrobial resistance gene	**mecA/C and MREJ** (Methicillin resistance)	Linezolid or Vancomycin	In association, in case of **septic shock or after prior exposure to broad-spectrum** beta-lactams (cefepime, carbapenems) in case of potentially **difficult to treat bacteria**
	**CTX-M** (ESBL)	Meropenem or Imipenem	
	**KPC, Oxa48-like*** (Carbapenemases)	Ceftazidime-Avibactam + Colistine	
	**NDM, VIM, IMP*** (Carbapenemases)	Meropenem or Imipenem + Colistine or Cefzazidime-Avibactam + Aztreonam	
Potentially difficult to treat bacteria	***A.baumannii***	Meropenem or Imipenem or Ceftazidime	
	E. cloacae	Cefepime	
	K. aerogenes	Cefepime	
	S. marcescens	Cefepime	
	P. aeruginosa	Meropenem or Imipenem or Ceftazidime	
Intracellular bacteria	***L. pneumophila***	Spiramycin + Levofloxacine	
	C. pneumoniae	Spiramycin	
	M. pneumoniae	Spiramycin	
Viruses	***Influenza A/B***	Oseltamivir + amoxicillin/clavulanic acid	
	**Others**	Nothing	
Positive for others	***Proteus sp***	Amoxicillin/clavulanic acid	
	K. pneumoniae	Third generation cephalosporin	
	K. oxytoca	Third generation cephalosporin	
	E. coli	Third generation cephalosporin	
	H. influenzae	Third generation cephalosporin	
	M. catarrhalis	Amoxicillin/clavulanic acid	
	S. aureus	Amoxicillin/clavulanic acid	
	S. group A or B	Amoxicillin/clavulanic acid (amoxicillin if no other bacteria is identified)	
	S. pneumoniae	Amoxicillin/clavulanic acid (amoxicillin if no other bacteria is identified)	
Negative for all bacteria and resistance genes tested **		Consider Gram stain examination :<br>- Nothing: Amoxicillin/clavulanic acid<br>- Gram-negative bacilli : consider ***S .maltophilia, M.***	

	morganii, C. freundii, C. koseri, H. alvei	

* Call the infectious team and test for Colistine, Tigecycline and Fosfomycine

**** Call the infectious team**

Table S2: Subgroup analysis of the primary outcome

	FAPP based treatment	Recommendations-based treatment			Real-life treatment		
Subgroups			difference with PCR	p Values	days	difference with PCR	p Values
Trauma (n=42)							
Broad spectrum, yes	36%	33%			22%		
Broad spectrum, days	0 [0, 0]	2[2, 3]	2[0, 2]	< 0.0001	0[0, 6]	0[0, 2]	0.003
Medical (n=32)							
Broad spectrum, yes	15%	37%			24%		
Broad spectrum, days	0 [0, 2]	0 [0, 0]	3 [2, 4]	< 0.0001	3 [0, 7]	0 [0, 0]	< 0.0001
Surgical / Other (n=26)							
Broad spectrum, yes	81%	67%			70%		
Broad spectrum, days	1[0, 2]	2[2, 3]	[-2, 0]	< 0.002	2[0, 5]	0[-2, 0]	0.01

Comparison of the number of days under broad-spectrum. FAPP: FilmArray® Pneumonia plus Panel.
